# Neuroborreliosis with encephalitis: a broad spectrum of clinical manifestations

**DOI:** 10.1186/s12879-025-10588-0

**Published:** 2025-02-07

**Authors:** Dominica Hudasch, Franz Felix Konen, Nora Möhn, Lea Grote-Levi, Ulrich Wurster, Gabriel Welte, Josef Conzen, Nima Mahmoudi, Thomas Skripuletz, Philipp Schwenkenbecher

**Affiliations:** 1 Department of Neurology, Hannover Medical School, Hanover, Germany; 2 Department of Neurology, Nordstadt Hospital Hannover, Hanover, Germany; 3 Department of Radiology / Neuroradiology, Nordstadt Hospital Hannover, Hanover, Germany; 4 Department of Diagnostic and Interventional Neuroradiology, Hannover Medical School, Hanover, Germany

**Keywords:** Lyme borreliosis, Encephalitis, Neuroborreliosis, CSF, CNS infection

## Abstract

**Background:**

Lyme neuroborreliosis is the disseminated stage of an infectious disease caused by Borrelia burgdorferi (BB). The most prevalent clinical manifestations include meningoradiculitis and involvement of the cranial nerves with lymphocytic meningitis. Facial nerve is the most frequently affected cranial nerve. Neuroborreliosis presenting as encephalitis is only scarcely described in the literature.

**Methods:**

Medical records of patients between 2014 and 2024 were screened and reviewed for neuroborreliosis. Patients were only included if they met the diagnostic criteria for definite neuroborreliosis.

**Results:**

A total of seven patients with neuroborreliosis induced encephalitis were identified. The median age was 72 years, and except for one man, all patients were women. Clinical manifestations ranged from cognitive impairments resembling primary dementia to movement disorders mimicking Parkinson's disease, tremor, and epileptic seizures. In 5/7 patients (71%) they occurred between August to September. In addition to central nervous system involvement, all patients exhibited characteristic features of neuroborreliosis like painful meningoradiculitis or peripheral facial palsy. An elevated cell count in cerebrospinal fluid (CSF), positive oligoclonal bands, and blood-CSF barrier dysfunction indicated by an elevated albumin quotient (QAlb) was found in all patients. Advanced age, in which immunosenescence can occur, might contribute in developing encephalitis caused by neuroborreliosis in the patients of this cohort.

**Conclusion:**

Neuroborreliosis can present with symptoms that mimic various neurological disorders, such as dementia, movement disorders, and epilepsy. Radiculitis, peripheral facial palsy or meningitis are an additional hint for this infectious disease. Analysis of CSF, which includes testing for intrathecally produced BB antibodies, is crucial for the diagnosis.

## Introduction

Lyme borreliosis is an infectious disease caused by the spirochete Borrelia burgdorferi (BB) sensu lato which is transmitted by tick bites [1]. After the tick bite, BB invades the skin and can result in a local infection which is called erythema migrans (EM). In the second stage of Lyme disease, the bacterium has the potential to disseminate hematogenous to various organs including the heart, joints, and the peripheral and central nervous system. BB uses different immune evasion strategies, including the inhibition of the complement system [2].

Neuroborreliosis can manifest in the peripheral and central nervous system with different clinical features [3–5]. One of the most common and pathognomonic manifestation for Lyme neuroborreliosis is the so called Bannwarth triad consisting of lymphocytic meningitis, peripheral facial palsy and painful radiculitis [1]. In cases of clinical suspicion, the diagnostic work-up consist of cerebrospinal fluid (CSF) examination and determination of Borrelia burgdorferi specific antibody in serum and CSF and their intrathecal synthesis [6]. A definite neuroborreliosis is then defined by evidence of CSF pleocyctosis and a BB specific antibody production within the CSF compartment. This intrathecal antibody production is calculated and displayed by the antibody specific index (ASI) [7]. However, the recognition of this neuroinfectious disease can be challenging since it can manifest in different stages and in numerous clinical characteristics [1, 8]. According to national guidelines early and late neuroborreliosis should be treated with doxycycline, ceftriaxone, cefotaxime or penicillin G [6].

Encephalitis, or inflammation of the brain parenchyma accompanied by neurological dysfunction, is often described in the acute state as a triad of symptoms, including headache, fever and altered mental status [9]. Magnetic resonance imaging (MRI) can show abnormalities typical of encephalitis and possibly provide information regarding the etiology [10]. However, CSF diagnostics is obligatory in the diagnosis of encephalitis [6].

Supplementary electroencephalogram (EEG) examinations can show epilepsy-typical potentials and thus support the diagnosis [9]. Since a wide range of infectious agents and autoimmune diseases can cause encephalitis, neuroborreliosis can be overseen resulting in misdiagnosis or delayed diagnosis [11].

Data about common MRI findings or typical EEG patterns in patients with neuroborreliosis induced encephalitis are scarce and appear rather as an anecdotal narrative or case reports than a structured exploration of these clinical manifestation.

The objective of this study was to provide a comprehensive analysis of the clinical features, CSF, MRI and EEG findings of patients with neuroborreliosis who presented with encephalitis. This analysis was conducted in order to raise awareness of this easily treatable neuroinfectious disease.

## Results

A total of seven patients with encephalitis due to neuroborreliosis were included in the study which were treated between 2014 and 2024. In this period, altogether 57 patients with neuroborreliosis were identified at our research center indicating that this cohort represents 12% of the neuroborreliosis cases. None of these patients had neurological symptoms and signs for more than 6 months like with late neuroborreliosis. The median age of all patients in this study was 71 years (IQR: 66–74 years) and six of the seven patients were women (86%).

All seven patients exhibited altered mental status for a duration exceeding 24 h, CSF pleocytosis, and evidence of an intrathecal immunoglobulin synthesis against BB indicated by an ASI > 1.4, thereby fulfilling the criteria for a definitive diagnosis of neuroborreliosis and for confirmed (4/7) or probable (3/7) encephalitis.

Only one patient reported a tick bite several months prior to admission. None of the patients reported an erythema migrans. No patient presented with fever (defined as a temperature above 37.9°C) at the time of presentation, and no signs of meningism were observed. The diagnosis of neuroborreliosis was established between August and January, with five patients being diagnosed between August and September. The initial indications were documented by patients or through third-party medical histories between the months of May and September. In five of seven cases, unspecific symptoms such as fatigue and general malaise were reported several months prior to the onset of neurological symptoms, which ultimately resulted in the patient seeking medical advisory. The interval between the initial manifestation of symptoms and the diagnosis of neuroborreliosis ranged from 10 days to 6 months.

One patient was diagnosed with diabetes mellitus as immunocompromising comorbidity. Two other patients had a history of malignant disease, which was either stable or cured at the time of diagnosis of neuroborreliosis.

All patients were tested with IgG-ASI > 1.4 against BB, and 4/7 patients were also tested positive for IgM-ASI. The results of simultaneous testing for Treponema pallidum infection in both serum and cerebrospinal fluid (CSF) were negative in all patients. Oligoclonal bands (OCB) restricted to CSF were found in all patients. A summary of the CSF findings and the antibody-specific indices for BB (IgM and IgG) can be found in Table 1.

**Table 1:** CSF findings of the first lumbar puncturing. Cerebrospinal fluid (CSF), CSF/serum ratio for albumin (QAlb), oligoclonal bands (OCB), antibody specific index (ASI), immunoglobulin G (IgG), immunoglobulin M (IgM)

Case no	CSF cell count (cells/µl)	CSF lactate (mmol/l)	QAlb	OCB	ASI IgG	ASI IgM
1	64	3.4	39.5	2	11.7	1
2	63	2.3	10.1	2	69.28	9.67
3	205	3.7	11	3	37.38	0.82
4	220	3.8	29.8	2	61.11	6.13
5	242	3.4	43.2	2	80.13	7.8
6	183	n.a	16.9	2	16.97	5.35
7	133	2.2	9.8	3	3.62	n.a

Other pathogens like syphilis, herpes simplex virus (HSV), varicella zoster virus (VZV), Epstein Barr virus (EBV), cytomegalovirus (CMV), enterovirus, and bacterial pathogens were tested negative.

**Fig. 1:**
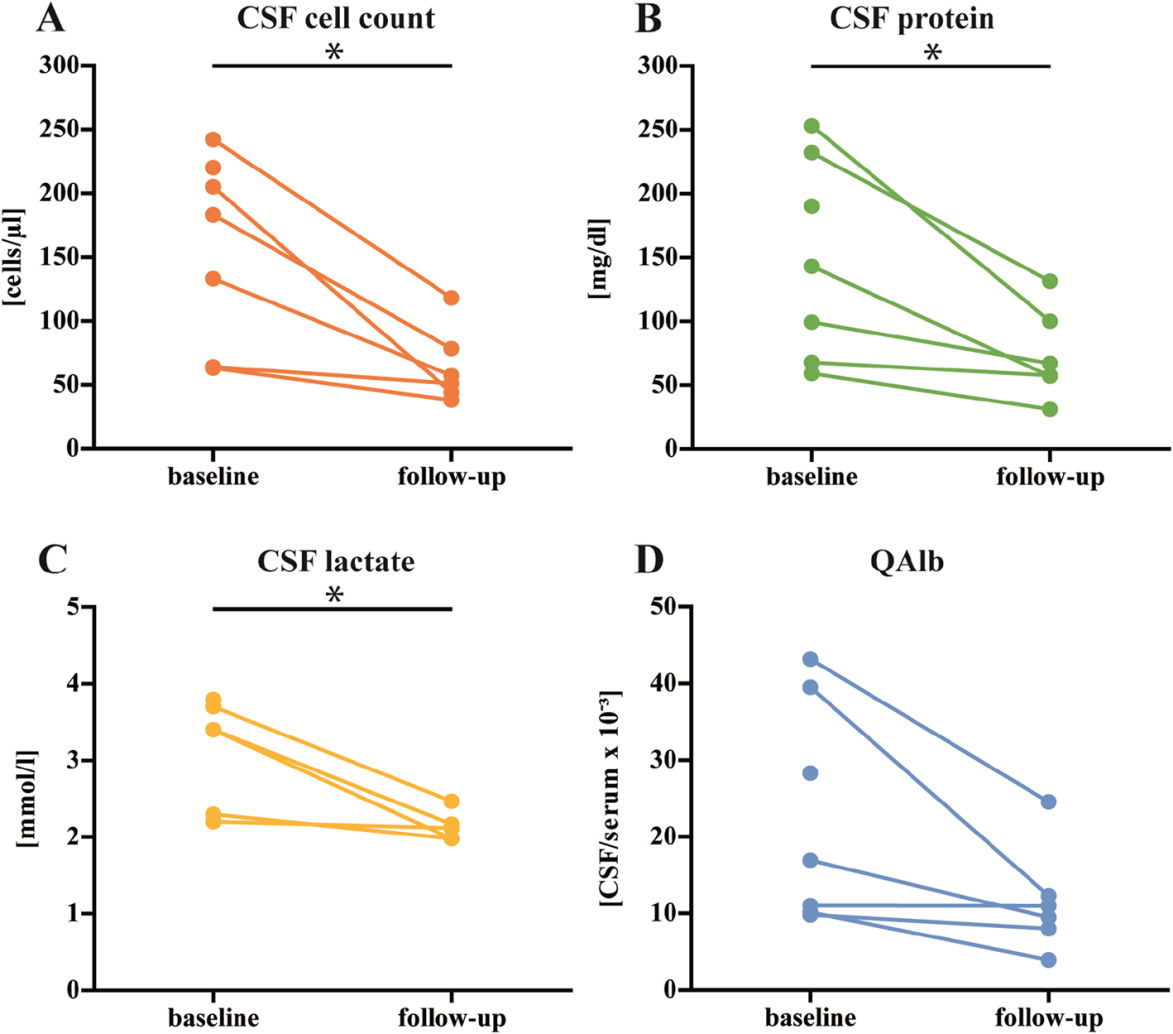
CSF findings of the first lumbar puncture and follow-up CSF examination on an average of 12 days after the initial CSF puncture. CSF cell count (**A**), CSF protein (**B**) and CSF lactate (**C**) decreased significantly under therapeutic treatment. (* indicated *p* < 0.05)

All patients received ceftriaxone intravenously following the diagnosis of neuroborreliosis. A reduction in the number of cells present in CSF was observed during the second week, accompanied by an improvement trend in the blood-CSF barrier function indicated by the CSF-serum albumin quotient (QAlb) in all patients under therapy (Fig. 1).

The following case descriptions will demonstrate the diverse range of encephalitis presentations associated with neuroborreliosis.

### Subacute dementia with inflammatory brain lesions (Case 1)

A 74-year-old woman presented with behavioral and personality changes that had been progressive for several weeks, compromising psychomotor retardation, lethargy and memory impairment. The patient reported headache of variable intensity and location for several months, in addition to an unintentional weight loss. The patient was diagnosed before with lichen ruber, autoimmune thyroiditis and a history of rectal cancer treated curatively with chemotherapy and radiotherapy one year ago with no evidence of recurrence.

She lived independently in her own home and had not exhibited any impairment of memory or loss of cognitive function thus far.

The neurological examination yielded evidence of dementia, as indicated by the patient's impaired memory and concentration.

**Fig. 2:**
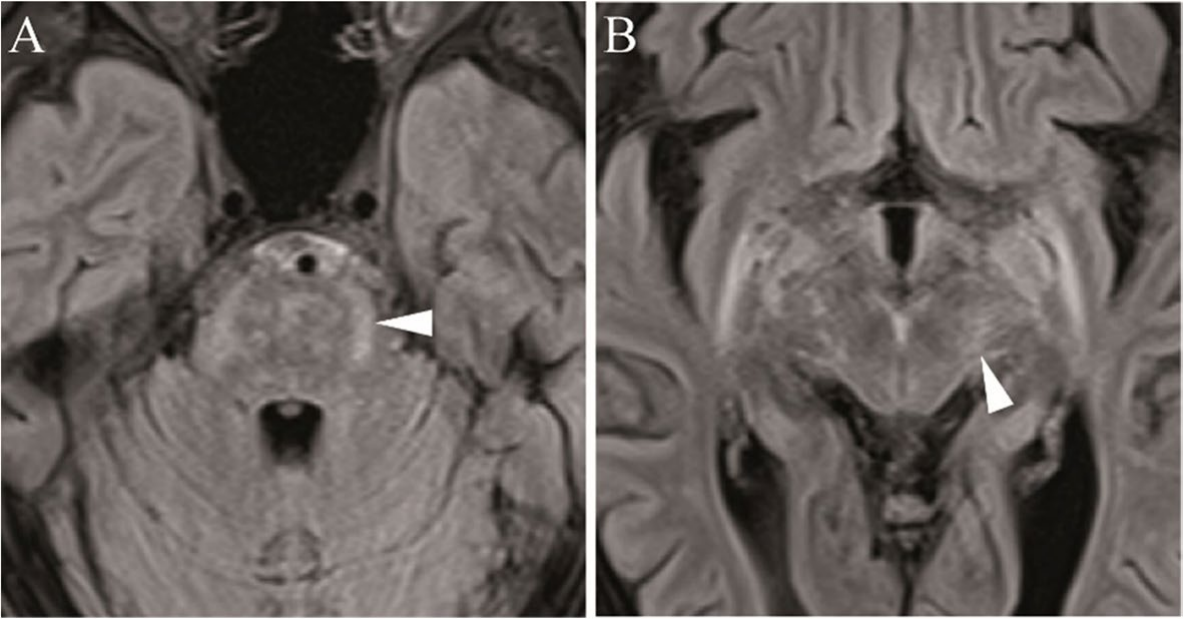
Magnetic resonance imaging (MRI) of the brain of Case A-B: Axial slices with fluid-attenuated inversion recovery (FLAIR), showing brainstem lesions spreading into the midbrain with an inflammatory appearance

An MRI scan of the brain showed lesions in the brainstem that resembled to be of inflammatory origin and had spread along the cerebral cortex to the thalamus (Fig. 2). EEG demonstrated intermittent rhythmic delta activity, which was most pronounced in the frontal lobes. A CSF examination revealed a lymphocytic pleocytosis (64 cells/µl), an elevated lactate concentration (3.4 mmol/l), a blood-CSF barrier impairment (QAlb 39.5), positive CSF-specific OCB, and intrathecal synthesis of immunoglobulins G against BB (IgG-ASI: 11.7)). A comprehensive differential diagnosis was performed, which excluded other CNS infections with syphilis, HSV, VZV, measles, rubella, and mumps.

The patient was administered ceftriaxone and exhibited clinical improvement throughout the course of treatment. The patient was later discharged without any evidence of neurological impairment.

### Frontal lobe syndrome with absence like episodes (Case 2)

A 76-year-old woman presented with a decline in consciousness, accompanied by short-term memory impairment, psychomotor retardation, pain and paresthesia in the right upper extremity for several weeks. Furthermore, relatives had observed recurrent episodes of absences. The patient was diagnosed with hypothyroidism and a sensorimotor polyneuropathy before. She lived in her own home without the need for assistance and had never previously exhibited any form of mental impairment. The neurological and neuropsychological examination (Consortium to Establish a Registry for Alzheimer's Disease (CERAD), Wisconsin Cart Sorting Test (WCST)) revealed an impairment in the transition from short-term to long-term memory and difficulties in executive functions, indicating the presence of a frontal lobe syndrome. An MRI scan of the brain demonstrated the presence of a generalized supra- and infratentorial symmetric pachymeningitis. EEG showed regional slowing, characterized by the presence of delta waves in the left temporo-occipital region. A CSF examination revealed an elevated cell count (63 cells/µl), an impairment of the blood-CSF barrier function (QAlb 10.1), and CSF-specific OCB. An intrathecal production of immunoglobulin G and M against BB was found (IgG-ASI: 69.3; IgM-ASI: 9.7). Other CNS infections with syphilis, HSV, VZV, EBV and CMV were excluded. The administration of ceftriaxone immediately alleviated both pain and paresthesia, while cognitive impairments also improved during the course of therapy, as confirmed by additional neuropsychological examinations.

### Acute delirium (Case 3)

An 84-year-old woman presented with acute onset of self-reported weakness and pain in her arms, accompanied by paresthesia in the left leg. The patient was diagnosed with type II diabetes mellitus, hearing loss and a breast carcinoma that had been treated with curative intent several years previously, with no evidence of recurrence. Despite her advanced age, the patient was living independently in her own home without the need for care. She was able to walk independently and was capable of organizing her everyday life. Neurological examination showed only a dysarthria with no evidence of paresis of the extremities but rather a pain-associated reduced mobility of the arms. MRI of the brain and EEG revealed no abnormalities. Three days after admission, the patient exhibited a rapidly progressive alteration in the level of consciousness. CSF analysis exhibited a pleocytosis with 205 cells/µl and an elevated lactate concentration (3.7 mmol/l), and a marked impairment of the blood-CSF barrier function (QAlb 11) with CSF-specific OCB and intrathecally produced immunoglobulin G against BB (IgG-ASI: 37.4). Other infections of the CNS with syphilis, HSV, VZV, EBV, CMV, and tick-born encephalitis (TBE) were excluded. The administration of ceftriaxone resulted in a swift recuperation of neurological symptoms, with only minor cognitive deficits remaining prior to discharge.

### Progressive cognitive decline in chronic psychosis (Case 4)

A 71-year-old woman presented with progressive disorientation and an exacerbation of memory impairment that had been ongoing for 6 months. The patient was previously admitted to the psychiatric ward since an organic psychosis was suspected. At that time, she exhibited episodes of acoustic hallucination. The patient presented with a history of coronary heart disease, arterial hypertension and hypothyroidism. In the neurological examination a peripheral facial palsy was found and neuropsychological testing revealed evidence of a dementia with predominant anterograde amnesia. An MRI of the brain did not exhibit any abnormalities. The EEG demonstrated generalized slowing. CSF examination showed a CSF pleocytosis (220 cells/µl), an elevated lactate level (3.8 mmol/l), a blood-CSF barrier impairment (QAlb 29.8), CSF-specific OCB, and intrathecal immunoglobulin G and M production against BB (IgG-ASI: 61.1; IgM-ASI: 6.1). Other CNS infections with syphilis, CMV, EBV, HSV, measles, and rubella were excluded.

Antibiotic therapy with ceftriaxone resulted in a slight regression of the peripheral facial nerve palsy, but there was no improvement in cognitive abilities.

### Parkinsonism (Case 5)

A 69-year-old women presented with a three-week history of neck pain, followed by fatigue, insomnia, behavioral change, memory impairment and decreased concentration, gait abnormality, tremor and eventually dysosmia. No other diseases were documented. Prior to the onset of symptoms, the patient was fully independent, requiring no assistance. She lived at home and participated in sporting activities without any gait impairment or tendency to fall. No evidence of cognitive disorders has ever been observed. The neurological examination revealed a bradykinetic gait disorder, which, in conjunction with an arm and head tremor, exhibited characteristics suggestive of Parkinsonism. An MRI scan of the brain demonstrated a non-contrasted enhanced lesion within the left thalamus. The EEG revealed no abnormalities. The CSF examination showed an elevated cell count (242 cells/µl), an elevated CSF lactate (3.4 mmol/L), a blood-CSF barrier function impairment (QAlb 43.2) and CSF-specific OCB. An intrathecal immunoglobulin G and M production against BB was found (IgG-ASI: 80.1; IgM-ASI: 7.8). Other CNS infections with syphilis, HSV, VZV, EBV and TBE were excluded. After treatment with ceftriaxone, the patient was discharged with no cognitive deficits, though a mild persistent tremor was noted.

### Movement disorder with extremity and head tremor (Case 6)

A 66-year-old woman presented with back pain and tremor affecting all extremities and the head for three weeks. A chronic herniated disc in the lumbar spine had previously been diagnosed. The patient was able to live independently in her own home without requiring any external assistance. With appropriate analgesia, the patient was able to move without the use of any assistive devices. Deficits in cognitive functioning had not, thus far, been identified. The patient reported about a circadian variation of symptoms, with an increase in severity towards the evening. Neurological examination showed a head tremor and a fine intention and posture tremor. MRI of the brain revealed no abnormalities and EEG showed frontal intermittent rhythmic delta activity pronounced on the right side. Analyses of blood sodium level (115 mmol/l) and urine sodium level (87 mmol/l) led to the diagnosis of syndrome of inadequate antidiuretic secretion (SIADH). Despite normalization of sodium levels, the patient exhibited progressive behavioral abnormalities, including anxiety and fluctuating orientation disorders. A CSF examination exhibited lymphocytic pleocytosis (183 cells/µl), blood-CSF barrier impairment (QAlb 16.4), and an intrathecal immunoglobulin G and M production against BB (IgG-ASI: 17; IgM-ASI: 5.4). CNS infection with HSV, VZV, EBV, TBE, or enteroviruses were excluded. Symptoms improved significantly after the initiation of antibiotic therapy with ceftriaxone and the patient was discharged without neurological symptoms.

### Focal epileptic seizures (Case 7)

A 67-year-old man presented with a history of focal epileptic seizures and holocranial headaches that had been occurring for several weeks. The patient had been suffering from arterial hypertension and hyperlipidemia for an extended period of time. Prior to the onset of symptoms, the patient was independent, without cognitive or motor impairments, and did not require assistance or care. Neurological examination revealed a paraparesis and symmetrically increased reflexes in the upper extremities and the neuropsychological testing cognitive impairment. An MRI scan of the brain demonstrated leptomeningeal contrast enhancement bilateral frontoparietal pronounced on the left side. The EEG examination showed an increased level of cerebral excitability with frontal accentuation on both sides. A CSF examination revealed an elevated CSF cell count (133 cells/µl), a blood-CSF barrier impairment (QAlb 9.8), and CSF-specific OCB. An intrathecal immunoglobulin G synthesis against BB was found (IgG-ASI: 3.6). Other CNS infections with syphilis, HSV, VZV, EBV and CMV were excluded. Following the commencement of antibiotic therapy with ceftriaxone, a rapid improvement in symptoms was observed. There was a residual increased reflex response and a positive Babinski reflex on the left.

## Material and methods

### Patients

The medical records of patients diagnosed with neuroborreliosis between 2014 and 2024 at Hannover Medical School were subjected to a comprehensive review. One patient from an academic teaching hospital of Hannover Medical School (Nordstadt Hospital Hannover) who was treated in that period was also included.

Patients were included in this retrospective study only if they met the criteria for definite neuroborreliosis and definite/possible encephalitis (see Table 2). Exclusion criteria were alternative possible causes of an encephalitis like another concurrent CNS infection (two patients) or a CNS autoimmune disease (one patient).

**Table 2:** Diagnostic criteria for Neuroborreliosis, as defined by the European Federation of neurological societies and diagnostic criteria for encephalitis according to the International encephalitis Consortium [7, 12]

Definitive Neuroborreliosis	Confirmed encephalitis	Possible encephalitis
Neurological symptoms suggestive for neuroborreliosis in the absence of other apparent causes of the focal deficits	**Major criterion (required):**	**Major criterion** (Change in character or personality, qualitative or quantitative disturbance of consciousness persisting > 24 h without any other identifiable cause) + **2 minor criteria**
	Change in character or personality, qualitative or quantitative disturbance of consciousness persisting > 24 h without any other identifiable cause	
**AND**	**AND ≥ 3 minor criteria:**	
CSF cell count ≥ 5 cells/µl	• The occurrence of new focal neurological deficits • New focal or generalized epileptic seizures that cannot be attributed to a previously diagnosed seizure disorder • Fever of ≥ 38°C 72 h before or after hospitalization • CSF cell count ≥ 5 cells/µl • Radiological evidence of pathological changes within the brain parenchyma, or encephalitis • EEG signs consistent with encephalitis	
**AND**		
Detection of intrathecal synthesis of antibodies against BB		

Given the retrospective nature of this study, only data collected during routine procedures were subjected to analysis.

Prior to inclusion, the clinical symptoms and signs attributable to neuroborreliosis and encephalitis were reviewed by a board-certified neurologist. Furthermore, clinical data, including age, sex, comorbidities, self-reported tick bites or erythema, time from symptom onset to diagnosis, disease course, including antibiotic treatment, and follow-up, were also documented. Where available, MRI and EEG findings were screened and registered.

Written informed consent was obtained from all patients or their legal representatives. The investigation was approved by the institutional ethics committee (reference number 1580–2012).

### Clinical findings

The pain described, which occurred in conjunction with the other symptoms, was classified as radiculitis due to the nature of the pain and its localisation [8, 13]. This is supported by the fact that the symptoms regressed very quickly under antibiotic treatment [1, 14].

The examination of cognitive deficits was based on a neuropsychological examination using Consortium to Establish a Registry for Alzheimer's Disease (CERAD), Wisconsin Cart Sorting Test (WCST) and Montreal Cognitive Assessment (MOCA). Dementia was defined decline in cognition that is significant enough to interfere with independent, daily functioning [15].

### Laboratory testings

CSF cells counts > 4 cells/µl were defined as pleocytosis. Concentrations of immunoglobulins of IgA, IgG and IgM and albumin in CSF and serum were measured nephelometrically. Blood–CSF barrier function was assessed by QAlb [16]. The CSF/serum ratio of IgA, IgG, IgM and albumin were calculated and the immunoglobulin ratios plotted against the albumin ratio according to Reiber’s diagram [16].

CSF-specific oligoclonal bands were identified by isolectric focusing and consecutive silver staining respectively immunoblotting in one case [17].

The presence of specific IgM and IgG antibodies against BB sensu lato was determined via enzyme-linked immunosorbent assay (ELISA) blots [5].

The ASI against BB, which indicates an intrathecal production of immunoglobulins IgG or IgM, was calculated by dividing the ratio of CSF Ig Borrelia/serum Ig Borrelia to CSF Ig total/serum Ig total [18, 19]. In cases of an overall intrathecal immunoglobulin production, the fraction of BB specific antibodies was calculated by dividing the CSF Ig Borrelia/serum Ig Borrelia ratio to Qlim. Qlim is defined as the maximum ratio for Ig CSF concentrations to Ig serum concentration [18]. Values of the ASI index exceeding ≥ 1.5 indicate the synthesis of specific antibodies in the central nervous system (CNS) [18].

### Statistics

We used Prism Software (GraphPad Prism 8, California, USA) for statistical tests, calculations and creating graphics. Two-tailed P-value of *p < 0.05 were considered significant and designated accordingly. Paired t test was used to compare continuous variables between two time points.

## Discussion

In this study, we observed seven patients with neuroborreliosis-induced encephalitis, who exhibited a wide range of clinical manifestations, from cognitive impairments that resembled primary dementia to movement disorders similar to Parkinson's disease and epileptic seizures.

The reasons why an infection with BB manifests as an encephalitis remains unclear. In our study, all patients were older than 65 years which implies gradual deterioration of the immune system, brought on by natural age advancement may play an important role in developing this kind of encephalitis. It has been described that clinical manifestation of neuroborreliosis is age-depending with a higher proportion of radiculitis in contrast to meningitis affecting younger patients more frequently [20]. In analogy in a study about varicella zoster, CNS manifestation was also found more often in elderly patients which is most likely explained by immunosenescence as the gradual deterioration of the immune system by aging [21]. Noticeably, all but one patient were women indicating another risk factor in addition to immunosenescence. In contrast, comorbidities like malignancy or autoimmune disease with immunosuppressant were negligible in this group.

Dementia as a dominant symptom of neuroborreliosis is extremely rare and only few cases are described in which cognitive impairment was initially misdiagnosed as rapidly progressing primary dementia [22]. The patients in our study had also a subacute disease course affecting different cognitive domains like learning and memory, attention, language and executive function. One patient’s neuropsychological assessment revealed findings resembling frontotemporal dementia. In another patient however, onset of cognitive impairment was acute and resembled a delirium which has also been described as a manifestation of neuroborreliosis encephalitis [5]. The diagnosis of neuroborreliosis was especially challenging in a patient with cognitive decline developing over a course of 6 month who was diagnosed with chronic psychosis before. The cognitive decline could have been easily misattributed to the psychiatric disorder and in this case only the mild peripheral facial palsy was the indication for CSF examination which eventually led to the right diagnosis.

One important observation of this study is that encephalitis due to neuroborreliosis does not cause isolated cognitive impairment but is always accompanied by characteristic features of neuroborreliosis like painful meningoradiculitis or peripheral facial palsy.

Since all patients were in their senium a preexisting underlying cognitive impairment unnoticed by the patient and relatives could not be completely excluded. The clinical onset, the course of the cognitive impairment and the responsivity to antibiotics however would rather indicate against a primary dementia. Long-term follow-up data are missing but would be helpful to identify which of these patients were later diagnosed with a neurodegenerative disease. In these cases, the neuroborreliosis would rather be a confounding factor to reveal underlying conditions.

Additional to cognitive impairment two patients presented with movement disorders. One patient exhibited prodromal signs of Parkinson's disease, including sleep disturbances, olfactory impairment, tremors, and gait abnormalities. Another patient presented with tremor affecting all extremities and the head. This presentation was initially misinterpreted as a primary movement disorder before admission.

In another patient, focal epileptic seizures were the primary presenting symptom. Yet another patient was considered to suffer from ictal activity manifesting as absence like episodes.

The diversity of clinical manifestations underscores the necessity of lumbar puncture for CSF examination including the identification of intrathecal antibody production against BB which paved the way to the accurate diagnosis of neuroborreliosis.

The presence of inflammatory changes in the cerebrospinal fluid, accompanied by pleocytosis and impairment of the blood-cerebrospinal fluid barrier, has been observed in both neuroborreliosis in general and in neuroborreliosis encephalitis. All patients included in this study exhibited these characteristics. In contrast to probable or possible neuroborreliosis encephalitis, a higher proportion of an elevated CSF lactate concentration was found, which occurred in 4/7 patients [20].

MR imaging of the brain is typically one of the initial diagnostic procedures during the evaluation of potential neurodegenerative, autoimmune or infectious neurological diseases. While patterns of brain atrophy may indicate a type of primary dementia, MRI of the brain in cognitive impairment due to neuroborreliosis usually shows non-specific white matter lesions mostly periventricularly [22]. In our study, a patient with a subacute dementia exhibited brain lesions which were similar to brainstem alterations described in neuroborreliosis in another study [23]. In another patient with cognitive impairment which resembled frontal temporal dementia, a meningeal enhancement was found while parenchymal lesions could not be detected. Additionally, meningeal enhancement was observed in a patient with a history of epileptic seizures while a patient with Parkinsonism exhibited a thalamic lesion. In three patients brain MRI showed abnormalities attributed to inflammation. However, no specific pattern could be identified that was consistently present in all abnormal images. In summary MRI is of primary importance in differential diagnosis and does not show typical finding like in herpes simplex encephalitis [24].Abnormalities were identified in the EEG in five patients. These abnormalities ranged from focal to generalized slowing and included one-off evidence of epileptic potentials. Thus, the majority of the EEG findings were conspicuous and showed a pathological character, but the findings were very diverse, so that no clear pattern can be identified. Like MRI imaging, the EEG is an important tool, primarily for clarifying differential diagnoses in the case of neuroborreliosis encephalitis.

Neuroborreliosis is often listed as a potential differential diagnosis for chronic inflammatory central nervous system (CNS) disorders such as multiple sclerosis (MS) or neuromyelitis optica spectrum disorders (NMOSD) [25, 26]. The observations of this study do not support the assumption that MRI findings of neuroborreliosis are similar to these of chronic inflammatory CNS disorders. This is consistent with the results of a previous study in which brain parenchymal lesions were also identified only infrequently [5].

The prevalence of encephalitis due to neuroborreliosis is not exactly known, but reported to be low according to an investigation in a Scandinavian neuroborreliosis cohort, with a rate of 3.3%. [27]. In a previous study conducted by our department, we observed a slightly higher prevalence of neuroborreliosis and encephalitis, with 12% of patients [5]. Notwithstanding the aforementioned discrepancies, encephalitis can be regarded as a rare manifestation of neuroborreliosis. Consequently, we postulate, that it may be readily overlooked.

One limitation of this study arises from its retrospective nature which precludes the possibility of determining the neurological status of the patients prior to infection with neuroborreliosis. Since all patients were in their senium preexisting neurodegenerative diseases could not be entirely discounted. Nevertheless, since all patients or their next of kin described a notable neurological decline over a defined period, encephalitis must be considered as the underlying cause. Furthermore, all patients improved following antibiotic treatment already after a short-period which is typically observed in cases of neuroborreliosis [28–30].

## Conclusions

Neuroborreliosis should be considered as a differential diagnosis in patients with various neurological presentations including cognitive impairment, movement disorders or epileptic seizures. Radiculitis or peripheral facial palsy in these patients may serve as a crucial additional indicator for a neuroborreliosis. MRI and EEG can provide useful information but CSF examination including the determination of intrathecal produced BB antibodies is the key diagnostic procedure.

## Data Availability

Data supporting the findings can be found in the Tables. Additional data extracted may be shared upon request.

## Abbreviations

CNS: Central nervous system
CSF: Cerebrospinal fluid
EEG: Electroencephalogram
ELISA: Enzyme-linked immunosorbent assay
Ig: Immunoglobulin
LNB: Lyme neuroborreliosis
MS: Multiple sclerosis
MRI: Magnetic resonance imaging
NMOSD: Neuromyelitis optica spectrum disease
OCB: CSF-specific oligoclonal bands
QAlb: CSF-serum albumin quotients
